# On the Relationship between Intrinsic Saliency and Implicit Learning of Apologetic Strategies: The Case of Taiwanese EFL Learners

**DOI:** 10.3390/ejihpe11040095

**Published:** 2021-10-19

**Authors:** Chin-Ting Liu, Yuan-shan Chen

**Affiliations:** Department of Applied English, National Chin-Yi University of Technology, Taichung 411030, Taiwan; jimbo@ncut.edu.tw

**Keywords:** noticing hypothesis, implicit learning, intrinsic saliency, apology, L2 pragmatics

## Abstract

The current study explored the relationship between the intrinsic saliency of apologetic strategies and the effects of implicit learning. Seventeen Taiwanese English as a Foreign Language (EFL) learners with intermediate proficiency participated in the experiment with a pretest on apology in the first week, a noticing session in the second week and a posttest in the third week. The results from the noticing session indicated that providing reasons (e.g., *taking on responsibility*/*explanation or account*) and *offer of repair* were more salient in input to those learners. Additionally, the use of the apologetic strategies *taking on responsibility* and *offer of repair* increased in the posttest. Taken together, the results indicated that higher degrees of saliency in input led to better implicit learning outcomes. The interplay between input saliency and explicit/implicit learning as well as the pedagogical implications were discussed.

## 1. Introduction

The roles of explicit and implicit learning in second language (L2) acquisition have evoked a large proportion of discussions in the literature. To better understand this issue, the definitions and the differences between explicit learning and implicit learning from Hulstijn [1] (p. 131) are first shown below:

*Explicit learning is input processing with the conscious intention to find out whether the input information contains regularities and, if so, to work out the concepts and rules with which these regularities can be captured. Implicit learning is input processing without such an intention, taking place unconsciously.*

Based on this definition, one critical distinction between implicit and explicit learning lies in the fact that the former involves the learners’ awareness of what is being learned while the latter does not (c.f., [2]).

Although several studies have argued that L2 acquisition is largely implicit [3,4,5], many studies have also pointed out that the implicit–explicit distinction is a matter of degree in a continuum scale [6,7] and the employment of implicit or explicit learning in L2 acquisition would depend on the nature of the linguistic elements to be learned [2,7]. One of the critical factors that would exert such an influence on the suitability of implicit or explicit learning is whether the particular element in input could be brought into the learners’ focal attention without external metalinguistic facilitation (e.g., instruction). According to Schmidt’s *Noticing Hypothesis* [8,9,10,11]) in order for input to be converted to intake, learners must notice the gap between their current interlanguage and the target language forms. In this view, the linguistic input that learners could attend to without additional facilitation would be suitable for implicit learning and the input that learners might not be able to notice without assistance would require explicit teaching [12].

In this connection, the saliency degree of the linguistic elements in input plays a crucial role in L2 acquisition [2,13,14,15,16,17,18,19,20]. For instance, Loewen, Erlam and Ellis (2009) examined whether the extensive incidental exposure to the third person *-s* would help students’ performances. The results indicated that the experimental group did not outperform the control group in the posttest. The authors argued that one of the reasons for the experimental group’s failure was that the third person *-s* had a low saliency in input. Those authors hence claimed that the *intrinsic saliency* of the target structure played an essential role in L2 implicit learning. This view was echoed by Ellis [16], who also asserted that some features were inherently more salient and were easier to notice than others. Similarly, DeKeyser [2] proposed that some structures had a higher level of *objective difficulty*—the inherent difficulty of the grammatical features of concern. The level of the objective difficulty was negatively correlated with the input saliency. That is, when the input was intrinsically more salient, the level of objective difficulty was lower.

Despite the fact that the implicit–explicit distinction is also applicable to pragmatics [21] and that noticing the salient features in input is crucial to L2 pragmatics acquisition [22], most studies targeting the intrinsic saliency of a given element focus predominantly on morphosyntactic structures. Only a few studies have discussed the relationship between pragmatic saliency and L2 acquisition. One of those studies was carried out by Fordyce [12], who investigated how 81 Japanese native-speaking undergraduates acquired English epistemic stance forms such as the cognitive verb *think*, the evidential verb *seem*, the modal adverb *maybe*, the modal verb *might* and the modal expression *in my opinion*. The students were further divided into the explicit instruction group and the implicit instruction group. Although the results from the writing productions indicated that the learners from the explicit instruction produced a larger variety of epistemic stance types in the posttest and delayed posttest than did the implicit instruction group, Fordyce noted that the saliency in input played a significant role in the performances of the implicit instruction group. Specifically, both the implicit and explicit instruction groups had similar gains in the use of evidential verbs in the posttest and the delayed posttest. The author stated that the implicit group’s better performances in evidential verbs resulted from the fact that the form–function mapping of that particular kind of epistemic stance was salient in input and that the participants might notice the usage without explicit support. Another study that probed into the issue between pragmatic saliency and L2 acquisition was conducted by Eslami, Mirzaei and Dini [23]. In the study, the authors explored the effects of explicit and implicit teaching on Iranian English as a foreign language (EFL) learners’ acquisition of requests. After 12 weeks of intervention, the participants from the explicit teaching group outperformed those from the implicit teaching group in the discourse completion task, although the latter group also outperformed the control group. The authors claimed that the instruction and the feedback provided to the explicit teaching group promoted the saliency of the form–function mappings in the target language, which, in turn, assisted the learners to develop their pragmatic competence.

To summarize, as a necessary and sufficient condition for L2 acquisition to take place, learners must notice the gap between their existing interlanguage and the target language [9,10,11]. In the meantime, the literature has pointed out that different linguistic features possess different degrees of inherent saliency, which further influences the possibility that the features could be implicitly noticed by L2 learners. However, in comparison to studies focusing on the intrinsic saliency of morphosyntactic structures, relatively little attention has been paid to the pragmatic features. Additionally, previous studies have rarely assessed the intrinsic saliency of a particular feature in question, but indirectly infer their degrees of saliency in order to justify the learners’ acquisitional patterns observed in the experiments. Therefore, it is of great necessity to directly explore the intrinsic saliency of a given pragmatic element.

To bridge the gap in the literature, the current study aims at investigating the intrinsic saliency of apologetic strategies based on the data collected from Taiwanese EFL learners and whether higher saliency would lead to better implicit learning outcomes. Olshtain [24] defines apology as “a speech act which is intended to provide support for the H (hearer) who was actually or potentially malaffected by a violation X. Hence the act of apologizing is face-saving for the H and face-threatening for the S (Speaker), in Brown and Levinson’s terms” (p. 156). The research on apology originates from the Cross-Cultural Speech Act Realization Project (CCSARP) [25]. The languages examined within the CCSARP for apologies include American English, Australian English, Canadian French, German and Hebrew, and it is surprising that a universal manifestation in strategy selection can be observed across these languages [24]. The five apologetic strategies identified in the CCSARP include—*Illocutionary Force Indicating Device* (*IFID*), *taking on responsibility*, *explanation or account*, *offer of repair*, and *promise of forbearance*. Although the developmental trajectory of the Taiwanese EFL learners’ apologizing strategies has been extensively documented (e.g., [26,27,28,29]), the degrees of intrinsic saliency among the different apologetic strategies remain unknown. The specific questions to be answered in the study are shown in (1):

(1) Research Questions
  - Research Question 1: Which apologetic strategies are more salient in input to the Taiwanese EFL learners?
  - Research Question 2: To what extent do the different degrees of saliency lead to the Taiwanese EFL learners’ implicit learning of apologetic strategies?

The results of the study not only shed light on the issue of linguistic saliency, but also bear critical pedagogical implications for L2 practitioners in that language educators could better understand what features in input require explicit promotion so that they could be noticed by learners and therefore acquisition would be more likely to take place.

## 2. Methods

### 2.1. Participants

The data reported in the current study were collected from 17 Taiwanese EFL learners in an institute in the southern part of Taiwan. The participants were native speakers of Mandarin Chinese, and they all possessed a score of 60 (out of 100) or above from a mock English Comprehension Level (ECL) test (Mean = 67.94; SD = 6.55), which was developed by the Defense Language Institute English Language Center (DLIELC) in the United States. The test has been administrated in nearly 120 overseas sites and the scores were used to evaluate if the EFL users had the required proficiency to attend training sessions in the United States. According to DLIELC student Profile Guidelines [30], individuals with an ECL score of 60 or above are able to write simple and complex sentences; to seek and give information, to request and grant permission, and to express their preferences. Therefore, these participants were deemed to have intermediate proficiency and were capable of performing the tasks to be described below.

### 2.2. Design, Materials and Procedures

**Figure 1:**
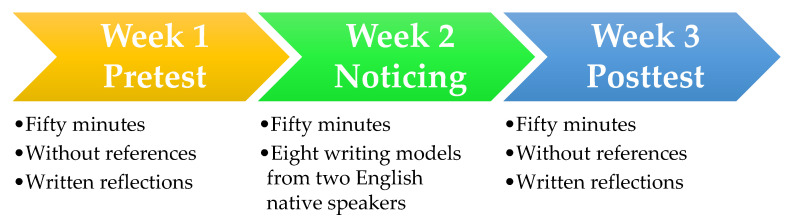
Design of the current study.

The current study employed a pretest–treatment–posttest design. The procedure is graphically shown in Figure 1.

For both the pretest and the posttest, the participants were told that they were the representatives of an international trading company and were responsible for checking and replying to the messages for the company’s official Facebook account. In one day, they found four private messages from different customers regarding mistakes with their orders. The participants were told that the company was liable for all the mistakes and that they had to respond to these messages. The complete prompt is shown in the Appendix A. For each test, the participants were required to finish replying to all the messages within 50 min and without checking any references.

The noticing activity was held in the second week. In this session, the participants received the pretest writing they had formulated in the previous week along with two writing models from two native speakers for each of the four situations in Appendix A, giving rise to a total of eight writing models for them to observe. The native speakers were instructed to include the five apologetic strategies listed in (2) below and one example of the sample writing is shown in (3). These five strategies were adapted from Blum-Kulka and Olshtain [31] and Blum-Kulka, House and Kasper [25]:

(2) The Five Apologetic Strategies
  - *IFID*:
    Expressions used to explicitly show speakers’ apology
    (e.g., I’m terribly sorry/I apologize for…/Pardon me for…).
  - *Taking on responsibility*:
    Expressions used to show the speakers’ responsibility for the offence
    (e.g., My mistake/I didn’t mean to upset you/I feel awful about it).
  - *Explanation or account*:
    Expressions used to explain that the violation at hand resulted from external factors and the speaker had no or little control
    (e.g., The traffic was terrible/The suppliers of the materials made a mistake).
  - *Offer of repair*:
    Expressions used to offer compensation for the damage, inconvenience or the loss
    (e.g., I’ll pay for the damage/A 30% discount is offered for your next purchase).
  - *Promise of forbearance*:
    Expressions used to promise that the offensive act will not happen again
    (e.g., This won’t happen again/I promise that the right order will be delivered to you on time in the future).

(3) One sample model used for the noticing activity
  (The brackets and the information in the brackets were not shown in the actual experiment.)
  [*Showing apology*] I’m really sorry for the shipping error, Mandy. [*Taking on responsibility*] I am at fault for not catching it earlier. [*Explanation*] The company recently switched to a new ordering system but it still has some bugs which caused the error in your order. [*Offer of repair*] Of course, I will send you the remaining 500 pairs express and will take 10% off as an apology. [*Promise of forbearance*] This won’t happen again.

The participants were required to note the differences between their own writing and the writing models they read. They were given 50 min to finish the activity.

### 2.3. Data Analysis

In order to examine which apologetic strategies were more salient in the input to the learners, the notes the learners took at the noticing stage were analyzed. One frequency was counted for an apologetic strategy when that apologetic strategy was noted by a participant. The percentage of the participants that noted each of the five apologetic strategies was counted as follows: Noticing frequency of an apologetic strategy/17 ∗ 100%. For instance, when 8 of the 17 participants noticed an apologetic strategy, the frequency count was eight and the percentage of noticing was 47.06% for that particular apologetic strategy (i.e., 8/17 ∗ 100%), but one point is appropriate to highlight here. Many written notes were not able to be neatly categorized into either *taking on responsibility* or *explanation or account*, because the participants used wordings such as *找藉口* (*zhǎo jiè-kǒu*, ‘finding excuse’) or *解釋原因* (*jiě-shì yuán-yīn* ‘explaining the reason’). Take *解釋原因* (*jiě-shì yuán-yīn* ‘explaining the reason’) for instance. The use of the term indicated that the participant noticed that a reason was generally provided in the writing models. However, as the participant did not specify if the reason referred to the speaker’s responsibility (i.e., *taking on responsibility*) or to external factors that the speaker had no or little control over (i.e., *explanation or account*), the term could not be neatly categorized. Therefore, these two categories were coded together as *providing reasons*. Although a distinction between *taking on responsibility* and *explanation or account* might not be transparent from the participants’ notes at the noticing session, the quantitative results from their posttest writings informed us greatly about which of these two apologetic strategies might possess a higher intrinsic saliency to the participants. We will return to this issue in the Discussion section.

The participants’ pretest and posttest writings were coded based on the criteria in (2). The number of the apologetic strategy types used in each of the four responses was coded and the number of each apologetic strategy type was summed for each participant, giving rise to a score ranging from zero to four for each apologetic strategy type. One major coder coded all the data and around 29% of the data (i.e., 5 out of 17) were coded by a second trained coder in order to establish the interrater reliability. Wilcoxon signed-rank tests, the nonparametric test equivalent to the paired-samples t-tests, were used to explore whether the differences between the number of each apologetic strategy used in the pretest and the posttest were statistically significant different.

## 3. Results

### 3.1. Frequency and Percentage of the Noticed Apologetic Strategies

In order to investigate which apologetic strategies were more salient in input to the Taiwanese EFL learners, the frequency and the percentage of the noticed apologetic strategies were calculated. The results are shown in Table 1 and some excerpts are shown in (4).

(4) Excerpts from the Noticing Stage (S = Student)
  - *IFID*:
    - *I didn’t think of using the term ‘apology for’ to apologize.*(S17).
  - Providing reasons (i.e., *taking on responsibility & explanation or account*):
    - *Factors contribute to the mistakes* (S5, S16).
    - *They provide reasons for the mistakes* (S6, S8, S10).
  - *Offer of repair*:
    - *I learn to provide discounts for them [the customers]* (S2, S3, S11).
    - *Offering discounts could be a way to apologize* (S13, S15).
  - *Promise of forbearance*:
    - *They [native speaker models] promise not to make the same mistake again* (S3).
    - *It becomes more persuasive when they [native speaker models] promise not to make the same mistake again* (S1).

The apologetic strategy that was the most salient in input to the participants was the *offer of repair*, with a total of 13 participants (76.47%) noticing the strategy. *Providing reasons* (i.e., *taking on responsibility* and *explanation or account*) was also salient to the participants, with a total of nine participants (52.9%) noticing the need for providing a reason. In addition, *promise of forbearance* was less salient than *offer of repair* and *providing reasons*. Finally, *IFID* was rarely reported by the learners when they were comparing their writings with the writing models.

### 3.2. Relationship between Noticing and L2 Apologetic Strategies

The Pearson product-moment correlation coefficient was used to test the interrater reliability of the two coders. The results showed that the coding reliability was strong (*r* = 0.895, *n* = 50, *p* < 0.001). The results of the average apologetic strategies used by the participants in the pretest and the posttest are shown in Table 2.

Statistical analyses using Wilcoxon signed-rank tests indicated that *taking on responsibility* and *offer of repair* were more frequently employed by the Taiwanese EFL learners in the posttest. According to Cohen [32] (p. 80) the effect sizes of these two apologetic strategies were large (i.e., *r* ≥ 0.5).

## 4. Discussion

The purpose of the study was to examine the intrinsic saliency of apologetic strategies and whether a higher saliency would lead to the Taiwanese EFL learners’ implicit learning. To this end, a pretest–treatment–posttest writing experiment was conducted, where 17 Taiwanese EFL learners were required to reply to Facebook messages regarding the complaints they received from customers in the pretest and the posttest. A week after the pretest, the participants were invited to compare the differences between their own writings and the writing models written by two native speakers of English. The results indicated that most of the participants noticed *providing reasons* (i.e., *taking on responsibility* and *explanation or account*) and *offer of repair*. The quantitative analysis echoed the results from the noticing stage in that the participants made statistically significant improvements in *taking on responsibility* and *offer of repair*. Taken together, the results indicated that higher degrees of saliency in input led to better implicit learning outcomes. Several related issues are discussed below.

The first research question asked which apologetic strategies were more salient in input to the Taiwanese EFL learners. The results from Table 1 indicated that *providing reasons* and *offer of repair* were the strategies that had higher degrees of saliency in the input to those learners. One intriguing point was that the learners generally ignored *IFID* in their noticing even though the expressions always appeared at the first sentence of each writing model. This observed phenomenon could be elegantly accounted for by Schmidt’s *Noticing Hypothesis* [8,9,10,11]. In this hypothesis, what is to be noticed is the *gap* between the learners’ interlanguage and the target language [9,10,11]. According to the scores from Table 2, the participants could employ an average of 3.059 (out of 4) *IFID* in the pretest, suggesting that they had acquired the knowledge and that such a gap was not existent. Therefore, it was reasonable that the learners did not note *IFID* in the noticing stage. In short, the intrinsic saliency of the apologetic strategies in input would have been influenced by the participants’ current interlanguage ability. When the strategies have been acquired, the saliency of the elements in input declines.

The second research question intended to explore the extent to which the differences in the intrinsic saliency of the apologetic strategies would lead to the Taiwanese EFL learners’ implicit learning outcomes. The results from Table 1 and Table 2 indicated that the higher intrinsic saliency of the apologetic strategies would lead to better implicit learning outcomes. Specifically, the learners noticed that expressions used to provide reasons for the mistakes and to offer repairs were generally used in the writing models. In the posttest, they produced statistically significant numbers of expressions used to show responsibility and to offer repairs. On the contrary, *IFID* and *promise of forbearance* were less salient in input; consequently, the numbers of the strategies employed in the posttest did not increase.

Given the strong connection between the saliency of the apologetic strategies in input and the effects of implicit learning, it is likely that the participants focused more on *taking on responsibility* rather than *explanation or account* at the noticing stage. That is, there was a noticeable improvement in the posttest uses of *taking on responsibility*, but this improvement was not found for the uses of *explanation or account*. One possibility for the results might derive from Taiwanese culture. According to Wang and Mattila [33], when there was a service failure, Taiwanese (in comparison to Americans) generally accepted a direct apology from the service provider because they value intragroup harmony and others’ face issues. Therefore, this might explain why *taking on responsibility*, which expresses the speakers’ remorse, might be more salient in input. To be precise, *taking on responsibility* is the apologetic strategy that the speakers and the listeners would expect in the given situation, leading to a higher possibility of being noticed in input.

The experimental findings provided essential pedagogical implications. Specifically, *explanation or account* and *promise of forbearance* had lower intrinsic saliency and were less likely to be noticed. Therefore, L2 practitioners need to deliberately increase the saliency of these strategies in input by providing explicit explanation, feedback or other techniques so that the learners’ awareness of, and attention to, those strategies could be brought into focal prominence. Another critical implication from the study was that understanding the intrinsic saliency of a target feature might be a prerequisite for efficient and effective language instruction. Given that the time devoted in L2 learning and instruction might not always be sufficient, language instructors might wish to allocate significantly more time on elements requiring explicit promotion. Additionally, without the understanding of the intrinsic saliency of the pragmatic strategy or feature of concern, language instructors might risk promoting inappropriate elements, giving rise to students insufficiently noticing the input of other elements with lower intrinsic saliency.

## 5. Conclusions and Future Directions

The current study explores the issue of pragmatic saliency in input by focusing on Taiwanese EFL learners’ acquisition of apologetic strategies. Results from the pretest–treatment–posttest experiment revealed that some apologetic strategies in input were intrinsically more salient than others. In addition, the participants increased their use of those salient apologetic strategies in the posttest, showing that the apologetic strategies with higher saliency could be more effectively acquired implicitly. On the other hand, the apologetic strategies that were less likely to be noticed in input required explicit teaching. The degrees of saliency in input would decline once the strategies had been previously acquired by the learners (e.g., *IFID*) because what they normally noticed in input were the *gaps* between their interlanguage and the target language.

There are some limitations in the current study, which could suggest the directions for future endeavors. First, the current study did not include the learners’ proficiency levels as a parameter. If the saliency of the apologetic strategies can change in accordance with the learners’ proficiency levels, future studies might need to include more than one proficiency group. It is expected that the apologetic strategies that are more salient in input might change depending on the proficiency levels. Next, to explain why there were increases in the use of *taking on responsibility*, we inferred that the intrinsic saliency of the apologetic strategies was influenced by the participants’ L1 culture. To be exact, it was proposed that, in comparison to *explanation or account*, *taking on responsibility* might be a better fit for Taiwanese culture. Future studies are suggested to conduct an empirical study directly addressing this issue.

## Figures and Tables

**Table 1:** Frequency and percentage of the noticed apologetic strategies.

Apologetic Strategies	No. of Participants Noticed (Max = 17)	Percentage (Max = 100%)
*Showing apology*	1	5.9%
*Providing reasons* *(Taking on responsibility &* *Explanation)*	9	52.9%
*Offer of repair*	13	76.47%
*Promise of forbearance*	7	41.18%

**Table 2:** Average number of the apologetic strategies used in the pretest and the posttest (Range: 0–4).

Apologetic Strategies	Pretest (SD)	Posttest (SD)	*Z*	*p*	Effect Size *r*
*IFID*	3.059 (1.197)	3.471 (0.717)	−1.461	0.144	−0.354
*Taking on responsibility*	0.765 (0.831)	1.647 (1.221)	−2.232	0.026	−0.541
*Explanation or account*	0.941 (1.088)	0.941 (1.144)	0	1	0
*Offer of repair*	2.647 (0.931)	3.353 (0.702)	−2.125	0.034	−0.515
*Promise of forbearance*	0.118 (0.485)	0.765 (1.300)	−1.841	0.066	−0.243

## Data Availability

The data presented in this study are available on request from the authors. The data are not publicly available due to original informed consent provisions.

## Appendix A

Replying to the Messages

**Situation**:

You are a sales representative of *Fine Shoes International Trading Corporation*. Today, you receive four private messages on Facebook from your major clients complaining about the products they received from the company. After seriously examining the packing and shipping processes, you realize that your own company is liable for all the mistakes. Now, you are about to reply to the four messages (as shown below) and inform the clients about the latest updates with regard to their complaints.

**Private Message 1:**
Hi, JB. Our company received the shoes yesterday. But there is a problem with the quantity. We ordered and paid for 1,000 pairs of sneakers but received only half of the products. Please reply ASAP.
Best, Mandy
**Private Message 2:**
Good morning, JB. Among the 500 pairs of slippers we ordered, 250 pairs should be size 7.5 (US sizing system). Unfortunately, all 500 pairs are size 6.5. Could you please check and write back to me soon? Regards, Ken
**Private Message 3:**
Dear JB: I just learned from my assistant that the shoes we ordered last month will arrive two weeks later than the timeline agreed to in the original plan. The late delivery will affect our marketing plans and our retailers will receive the goods later than we had expected. Sincerely, Brian
**Private Message 4:**
How are you, JB? Long time no see. I am writing to inform you about the mistake pertaining to the color of the shoes (Order No.: X89112a). We received red shoes last week, although we ordered blue ones. I’d like to touch base soon to determine what to do next.
Best regards,
Rita
